# Supplementary material for: Tead1a Initiates Transcriptional Priming Through the TEAD1a/YAP‐Notch1‐Spi1/Cebpα Axis to Promote Neutrophil Fate
Source: Adv Sci (Weinh). 2025 Aug 28;12(41):e05441. doi: 10.1002/advs.202505441 (PMC12591127; doi:10.1002/advs.202505441)
Supplement: Supplementary file 4 — Supplementary Dataset [file ADVS-12-e05441-s004.zip › 3-2.zip!/3-2/02-2.cluster/cluster4/cluster4_featureplot.pdf]

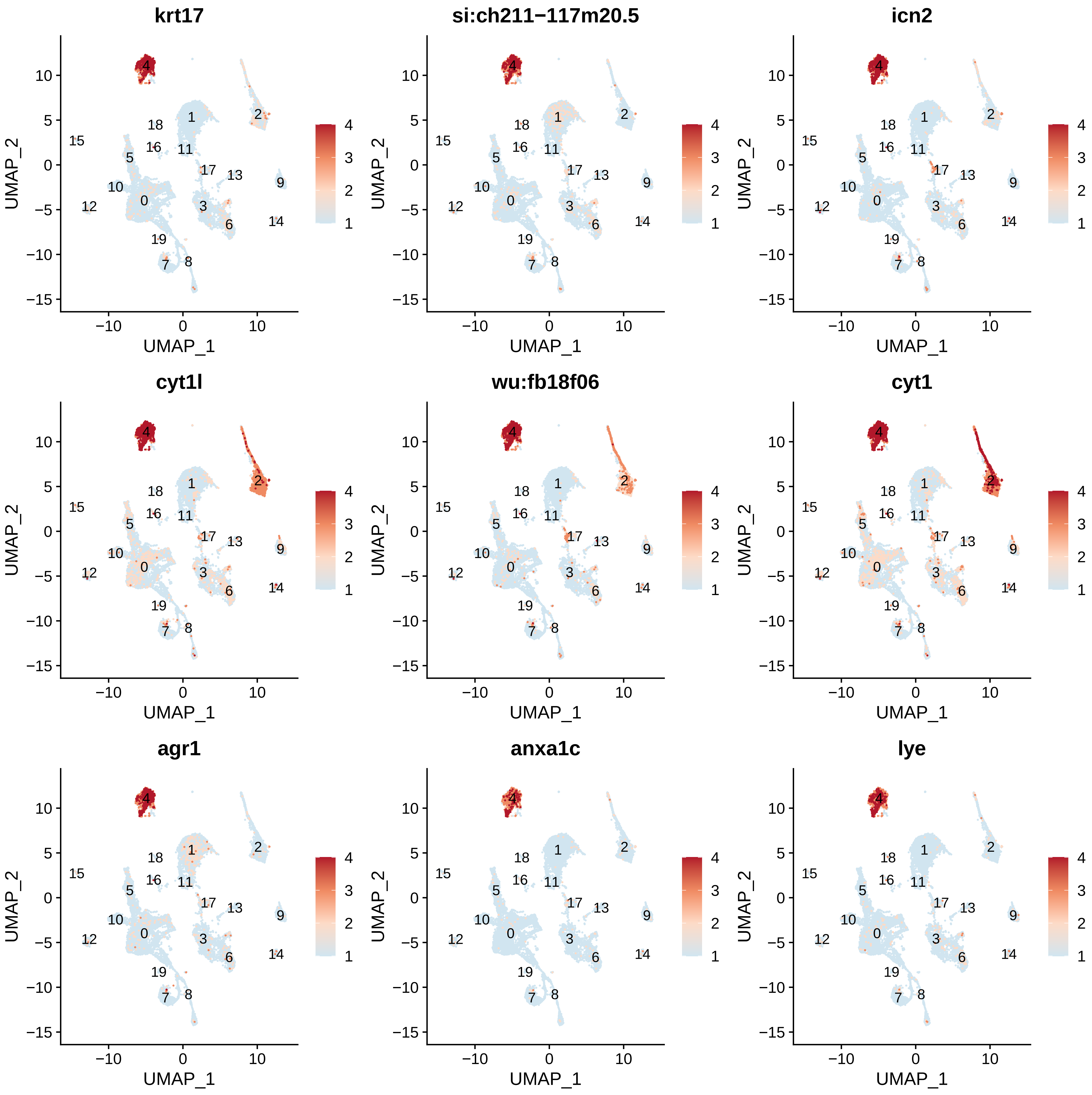
krt17
si:ch211−117m20.5
icn2
cyt1l
wu:fb18f06
cyt1
agr1
anxa1c
lye
UMAP_1
UMAP_2
−15
−10
−5
0
5
10
1
2
3
4
0
1
2
3
4
5
6
7
8
9
10
11
12
13
14
15
16
17
18
19
